# Remarks on the Early History of Medicine in America1Read at the annual meeting of the Ontario Medical Association, held at Niagara Falls, Ont., May 31, 1911.

**Published:** 1911-07

**Authors:** Roswell Park

**Affiliations:** Buffalo, N. Y.


### Remarks on the Early History of Medicine in America[1]

By ROSWELL PARK, M.D.
Buffalo, N. Y.

SUCH knowledge of medicine as obtained in this continent in the early days of the modern history of America was for a long time merely the filtrate, or that which had passed through the sieve of knowledge in the European centers of learning. This country having no great institutions of learning in which to furnish the desired knowledge it was necessary to go to the expense, the effort, even the hazard, to say nothing of the time required, of the trip to Europe, before one could acquire, if he would rise above the common level, anything more than elementary teaching.

During the century preceding the American revolution very few men had enough of these necessaries to permit such expenditure. It is not strange then that in America the same ignorance and superstitions prevailed that were to be met with only in the remoter parts of Europe. The scepticism of Montaigne and its final reduction by Bayle to universal doubt or universal credulity, or the mystical philosophy of Böhme, including the researches of Pascal and Malebranche, represented the then prevailing systems and methods of thought. Bacon had lived and died (in 1626) still spurning the discoveries of Copernicus. Hooke had founded the cell doctrine. Kepler and Gallileo had been persecuted and thus compelled to leave the verification of their discoveries to others, while Newton was born, only seven years after the first voyage of the Mayflower in 1642.

Academies of Science had sprung up in different parts of Europe, and the British Royal Society had been remodeled by Charles II, in 1662. In spite of these facts all kinds of superstition prevailed, alchemy flourished, witchcraft was in full bloom, and domestic animals were kept under cover during an eclipse in order to guard them against pestilences of unknown character.

Harvey had published his discovery of the circulation in 1628, and the microscope had been invented but a few years previous to this. Logarithms were not invented (by Napier in 1700) until the American colonies were nearly one hundred years old. Previous systems in medicine had been modified, although the systems of Van Helmont, of Sylvius and of Willis were still more or less in vogue. The great Sydenham appeared upon the scene about the time of the first voyage of the Mayflower, and until his death, in 1689, most of the English physicians who crossed the ocean had been more or less under the influence of his teachings.

1. Read at the annual meeting of the Ontario Medical Association, held at Niagara Falls, Ont., May 31, 1911.

The seventeenth century, even in this country, is important in the history of surgery since it witnessed the gradual subsidence of superstition and its displacement by inductive methods of thought and improved habits of observation. Thus, even among the Dutch, who settled many new colonies in this country, surgery gradually assumed a dignified position; while among the English, French and Germans it had attained to something still better. But these were still the days of the barber surgeons, who were not considered gentlemen and who were mainly not to be considered fit to associate with gentlemen, constituting, as they almost always did, an uneducated and illiterate class. The ban of the church still condemned those who took part in any operation accompanied by the shedding of blood. The worst possible conditions prevailed in Spain at the time when the early adventurers or pilgrims from that country landed upon our shores and began to make American history.

It must be confessed that at this time there was little here to attract travelers, except the spirit of adventure which educated men seemed to possess in minor degree. Many of the earlier physicians in the colonies were church deacons or politicians or both. Thus Samuel Fuller, who came to Plymouth in 1620, preferred to be known as a deacon rather than as a doctor, although he practised medicine faithfully and as wisely as anyone could in those days. John Winthrop, Jr., was not only a physician but a statesman, becoming Governor of the New Haven colony in 1667, and being known in England as one of the founders of the Royal Society. Up to 1692 one hundred and thirty-four men had been catalogued as practising medicine in the colonies. It constitutes a curious commentary on the times that the first person executed for witchcraft was one Margaret Jones who was also a physician.

The English colonists usually brought their physicians with them. Thus, when Jamestown, Virginia, was founded, in 1607, we hear of one Wotten, or Woolton, who came out as surgeon of the London Company, and who was considered by its members to be a gentleman. Even in those early days it was found necessary to pass laws regulating the practice of medicine, since quackery flourished to a considerable extent throughout the colonies.

In the Netherlands, at first controlled by the Dutch West India Company, it was a provision of their Charter that they should procure a "Comforter for the sick."

An item of exceeding great interest is to the effect that, in1772, one George Fox, a physician, was traveling with John Jay when the latter was thrown from his horse and had his neck apparently broken. Fox at once instituted a manipulation by which he apparently reduced the dislocation of the upper vertebrae. Whether this was what he actually accomplished or not we may only conjecture; but at all events Jay's life was restored. This is one of the earliest cases of this kind on record; in fact, if we assume its authenticity or correctness, it is in all probability the first case of dislocation of the bones of the spine reduced by manipulation. Viewed in this light it must be historic and memorable.

During that seventeenth century this country was scourged by epidemics of yellow fever, smallpox, scurvy, dysentery and many other diseases. About one hundred people started on the voyage to the west with William Penn, but one-third of that number perished of smallpox during the voyage. All along the coast smallpox prevailed and was not successfully combatted until Dr. Boylston dared to institute that method of inoculation to which his attention had been called by Cotton Mather, who had read of its successful introduction from Constantinople into England by Lady Mary Wortley Montague. This constitutes one of the most interesting episodes in the history of medicine in this country. The Rev. Cotton Mather, distinguished both as a politician and divine, still had this matter on his mind even after he had lost his interest in the burning of witches. In 1721 he had somewhere read a paper on "Turkish Inoculation," written by one Timmonius, and endeavored to transfer his interest to others, especially, as he hoped, to one Dr. Douglas. On failing with him, he turned to Boylston, then in Brookline, Mass., who appreciated both the importance of the method and the ripeness of the occasion; but so soon as Boylston's purpose became understood he was, as usual, denounced in the pulpits and attacked by the multitudes, and had as his only backer Mather, who had not yet lost his authority with the clergy nor failed in his support by them. Opposed, then, by colleagues, by the clergy and by the universal rabble, Boylston had the hardihood to inoculate first his son, and then two negro servants, only six weeks after the first inoculation had been done in London, by Mr. Maitland, for Lady Montague. The method was then and thus introduced into this country. Nevertheless, Boylston lived to reap both glory and profit from his intrepidity. The controversy which he aroused had well-nigh subsided when he went to London. There he found it still raging and in its defence he again aroused a storm but eventually triumphed. (It must be remembered that this was the Turkish method of actual inoculation of the disease by pus or discharge from the lesions of a patient suffering from genuine variola.)

Remembering the almost constant warfare between natives and new comers to this country, or among the latter between themselves, the scene of conflict extending from Quebec on the north to this very Niagara Frontier on the west, and to Georgia on the south, the Indians being active in some way nearly all the time, while the French and the English fought intermittently, one would imagine, considering these facts, that with the many opportunities thus afforded for the study of military surgery no small advance would be made in that science; but this does not appear—nothing was accomplished in the way of improvement or discovery, and the wounded soldier of 1776 had but little better treatment than the wounded pilgrim of 1676.

A few men stand out so prominently that they must needs be mentioned whenever the medical history of this country is under consideration. Among these were Colden, of New York State, who was born in Scotland in 1688 and came to this country in 1707. In 1710 he moved to Philadelphia and there wrote some of the first medical papers ever penned in this country. Later he took up his residence in New York, attaining remarkable popularity in practice, filling numerous public offices and preserving his reputation as an indefatigable student. He acquired a large estate and became the intimate friend of Benjamin Franklin, to whom he first suggested the foundation of the American Philosophical Society.

As the colonies increased in wealth and size Charleston became the more prominent center of influence, and here lived and died during the seventeenth century a group of five men who achieved unusual distinction and made important contributions to science. These men were Chalmers, Lining, Gardiner and Moultry, who were all born in Scotland, and Bull who was the first native of South Carolina to receive a doctor's degree. So high a place did Bull attain in public esteem that his death, after forty years of phenomenal activity, was regarded as a public calamity.

An important epoch in medical history of the eighteenth century was the foundation of the first hospital in this country. This must be credited to the energies of Benjamin Franklin and Drs. Bond, Shippen and Morgan. By their combined influence and efforts the way was prepared for the opening of the Pennsylvania Hospital in 1752. For four years they occupied rented quarters, then moved into a building of their own. Thus began an institution which had an enormous influence in more than one respect since, by its foundation, there was made possible the creation of the first medical school in this country, which became a part of the University of Pennsylvania, and which owed its inception to the study and liberal enthusiasm of John Morgan. Although Harvard College was founded in 1636, and Yale University in 1701, the medical department of Pennsylvania's University was in fact the pioneer medical school in the United States. Among the men most prominent in its early history was John Morgan who began his study during the period of the French and Indian wars. Coming into contact with the foreign surgeons, who came over with the English troops, he found his own ability far from commensurate with his ambitions. In consequence he spent, altogether, five years in foreign study; at first under the Hunters in London, and under Cullen and the Monroes in Edinburgh, then upon the Continent, particularly with Morgagni, in Padua. On his return he became closely associated with the younger Shippen, who belonged to the well-known medical family notable alike for liberal education and social distinction. This Shippen became, in fact, the first professor of surgery in this country, about 1765. A little later the faculty was joined by Rush, who became the most conspicuous figure of his day as a professional man. He was but thirty-one years of age when he became one of the signers of the Declaration of Independence. But the gathering war-clouds of the Revolution lessened the enthusiasm of all medical teachers and the work of carrying on a hospital and medical school devolved mainly upon the shoulders of three or four men.

An equally prominent figure in the history of New York City was Samuel Bard who, returning from a period of foreign study, was inspired with the thought of founding a medical school. To this end he associated with himself Clossy in anatomy, John Jones in surgery, Middleton in physiology, Rush in chemistry and Tennent in obstetrics, while he, himself, took charge of both surgery and practice. These men founded the Medical School of Kings' College. This faculty rather went to pieces after the British occupation of New York City, but three years after the war the college went on under the name of "Columbia" although with poor success. A revival of college interests was begun in 1792, while in 1807 the New York University opened a medical college which was soon disrupted by jealousy and strife among its faculty. Finally, in 1811, the two schools united under the name of the younger, and thereafter the College of Physicians and Surgeons prospered, later coming again under the aegis of Columbia. Meantime under Bard's influence the New York Hospital had been founded, in 1668, and chartered in 1770. It was in its new building that the Provincial Congress met during the second year of the Revolution; but on the arrival of the British the hospital was turned into a barracks and Bard and Jones joined the Continental armies. It was not until 1791 that the hospital was fully and finally equipped and entered upon its career of usefulness.

Such was the state of affairs at the time of our Revolutionary War. The science of military surgery had been scarcely yet created, even among the continental armies, and the barber surgeons still had a place. It was during the latter part of the war, when especially the French sent some of their best men to this country, that our raw and untrained army surgeons came in contact with a better class of men by whom they were, to some extent, inspired. But both the colonial and general governments dealt so stingily with their medical departments that the equipment, like the training of the medical men, was most meagre. Among the heroes of that time were the brothers Joseph and John Warren, of Massachusetts, the former of whom started Paul Revere upon his famous ride. He was made president of the Provincial Congress, and just before the battle of Bunker Hill was made Major-General of the Continental forces, preferring this office to that of Physician-General which he had been offered. During the battle he played the hero's part and with musket in his hand, fighting as would a private, was shot dead just at the end of the battle. The younger brother, John, lived to achieve fame and transmit it to a posterity who have well preserved it.

The first Surgeon-General of the Continental army was Benjamin Church, of Boston, who at first gave great promise of efficiency, but who was later detected in correspondence with the enemy, was then court-martialed, imprisoned for a year, then permitted to escape, to be lost at sea. His place was taken by John Morgan, of Philadelphia, already mentioned, who had more trouble in fighting the politicians than in fighting the enemy. Morgan was succeeded by his old associate Shippen, under whose guidance the medical department was at last conducted with dignity and benefit to all concerned.

Were we to follow up the whole of the period between the revolution and our great civil war it would be necessary simply to recount to you names which are doubtless familiar to everyone present. In such a list would prominently appear such men as Physic, McDowell, and Mott, of whom it was said that he tied more great vessels than any surgeon living or dead. Dudley, of Kentucky, would also be included in this list, which I would not make longer for fear of encroaching both upon your time and your interest. Nevertheless, I can but feel, in closing this most brief epitome, that the great men of Canada should be included with those of the States, giving credit to men rather than to political considerations or boundary limitations. Ours is a science which makes distinctions neither of race, locality nor creed; which recognises genius and ability as having neither country nor nationality, but as being God's gifts, which He has distributed without partiality, and which it is our duty to recognise as such and for which we should always be grateful, no matter where found.

430 Delaware Avenue.

